# Tetra­aqua­bis(3-fluoro­pyridine-4-carboxyl­ato-κ*N*)zinc(II) dihydrate

**DOI:** 10.1107/S1600536810003284

**Published:** 2010-02-03

**Authors:** Jonetha Fleming, Jennifer Kelley, LeRoy Peterson, Mark D. Smith, Hans-Conrad zur Loye

**Affiliations:** a Chemistry Department, Francis Marion University, Florence, South Carolina 29502, USA; b Department of Chemistry and Biochemistry, University of South Carolina, Columbia, South Carolina 29208, USA

## Abstract

In the title compound, [Zn(C_6_H_3_FNO_2_)_2_(H_2_O)_4_]·2H_2_O, the Zn^II^ atom is octa­hedrally coordinated in a ZnO_4_N_2_ environment by two 3-fluoro­pyridine-4-carboxyl­ate (3-fpy4-cbx) ligands and four water mol­ecules. The [Zn(3-fpy4-cbx)_2_(H_2_O)_4_] mol­ecules form a three-dimensional network through strong O—H⋯O and weak O—H⋯F hydrogen bonds between 3-fpy4-cbx and water mol­ecules. The crystal used for data collection was a twin, with the twin law corresponding to a 180° rotation about the real-space [001] axis. The major twin fraction refined to 0.795 (1).

## Related literature

For metal-organic compounds with ligands containing both pyridyl and carboxyl­ate donor groups, see: Ellsworth *et al.* (2008 ▶); Erxleben (2003 ▶); Wang *et al.* (2006 ▶). For specific properties exhibited by related metal-organic compounds, see: Chen *et al.* (2009 ▶); Evans *et al.* (1999 ▶); Xie *et al.* (2008 ▶). For typical Zn—O and Zn—N bond distances in similar metal-organic compounds, see: Wang *et al.* (2006 ▶).
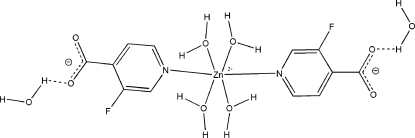


## Experimental

### 

#### Crystal data

[Zn(C_6_H_3_FNO_2_)_2_(H_2_O)_4_]·2H_2_O
*M* *_r_* = 453.65
Monoclinic, 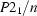

*a* = 6.6042 (4) Å
*b* = 19.1953 (10) Å
*c* = 6.8697 (4) Å
β = 99.225 (1)°
*V* = 859.61 (8) Å^3^
*Z* = 2
Mo *K*α radiation
μ = 1.51 mm^−1^
*T* = 294 K
0.28 × 0.22 × 0.16 mm

#### Data collection

Bruker SMART APEX CCD diffractometer
Absorption correction: multi-scan (*TWINABS*; Bruker, 2003 ▶) *T*_min_ = 0.890, *T*_max_ = 1.000
1734 measured reflections
1740 independent reflections
1605 reflections with *I* > 2σ(*I*)
*R*_int_ = 0.031

#### Refinement

*R*[*F* ^2^ > 2σ(*F* ^2^)] = 0.023
*wR*(*F* ^2^) = 0.065
*S* = 1.07
1740 reflections
149 parameters
H atoms treated by a mixture of independent and constrained refinement
Δρ_max_ = 0.28 e Å^−3^
Δρ_min_ = −0.19 e Å^−3^

### 

Data collection: *SMART-NT* (Bruker, 2003 ▶); cell refinement: *SAINT-Plus-NT* (Bruker, 2003 ▶); data reduction: *SAINT-Plus-NT*; program(s) used to solve structure: *SHELXS97* (Sheldrick, 2008 ▶); program(s) used to refine structure: *SHELXL97* (Sheldrick, 2008 ▶); molecular graphics: *DIAMOND* (Brandenburg, 2008 ▶); software used to prepare material for publication: *SHELXTL* (Sheldrick, 2008 ▶).

## Supplementary Material

Crystal structure: contains datablocks global, I. DOI: 10.1107/S1600536810003284/jj2018sup1.cif

Structure factors: contains datablocks I. DOI: 10.1107/S1600536810003284/jj2018Isup2.hkl

Additional supplementary materials:  crystallographic information; 3D view; checkCIF report

## Figures and Tables

**Table 1:** Selected bond lengths (Å)

Zn1—O3	2.0953 (14)
Zn1—N1	2.1356 (13)
Zn1—O4	2.1504 (13)

**Table 2:** Hydrogen-bond geometry (Å, °)

*D*—H⋯*A*	*D*—H	H⋯*A*	*D*⋯*A*	*D*—H⋯*A*
O3—H3*A*⋯O1^i^	0.83 (3)	1.86 (3)	2.6892 (18)	174 (2)
O3—H3*B*⋯O5^ii^	0.74 (2)	2.01 (2)	2.745 (2)	176 (2)
O4—H4*A*⋯O2^iii^	0.79 (3)	2.05 (3)	2.837 (2)	174 (2)
O4—H4*B*⋯O5^iv^	0.82 (2)	1.95 (3)	2.7643 (19)	171 (3)
O5—H5*A*⋯O2	0.75 (3)	2.05 (3)	2.796 (2)	174 (3)
O5—H5*B*⋯O1^v^	0.79 (2)	1.96 (3)	2.738 (2)	167 (2)
O5—H5*B*⋯F1^v^	0.79 (2)	2.55 (2)	2.9929 (17)	117.2 (18)

Symmetry codes: (i) 
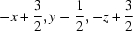
; (ii) 
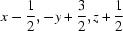
; (iii) 
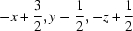
; (iv) 
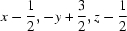
; (v) 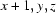.

**Fig. 1.**
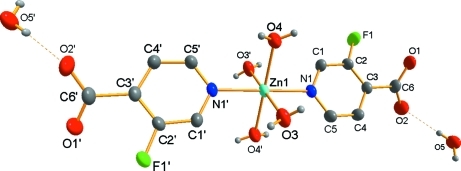
The molecular complex plus lattice waters with atom-labeling scheme of (I) showing 50% probability ellipsoids for nonhydrogen atoms. All H atoms except for those of water are omitted for clarity. Hydrogen bonds are represented by dashed lines. Primed atoms are generated by the inversion symmetry operation about Zn(II), with symmetry code: 1 - x, 1 - y, 1 - z.

**Fig. 2.**
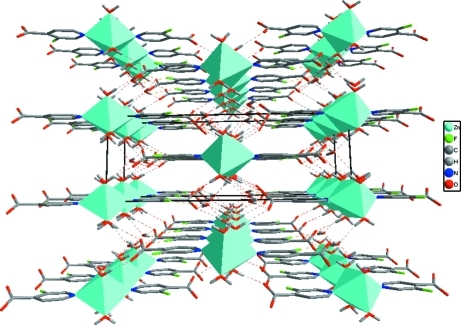
Wireframe polyhedral view of the crystal packing in (I) showing the hydrogen bonding scheme. Hydrogen atoms except for those of water have been omitted for clarity. Hydrogen bonds are represented by dashed lines.

## supplementary crystallographic information

### Comment

Metal-organic compounds based on multifunctional ligands that contain both pyridyl and carboxylate donor atoms have been under study in part because of their diverse coordination modes and because they may exhibit useful properties (Ellsworth *et al.*, 2008; Erxleben, 2003). Within this context, the 3-fluoropyridine-4-carboxylate ligand (3-fpy4-cbx), has attracted our interest as a potential component for the construction of these novel materials. A further motivation is that its nonfluorinated analogue, the isonicotinate ligand (ina), has been successfully utilized to generate metal-organic frameworks having the desirable properties alluded to above (Chen *et al.*, 2009; Evans *et al.*, 1999; Wang *et al.*, 2006; Xie *et al.*,2008). Hence, we have deemed it worthwhile to also explore the coordinatingproperties of the related 3-fpy4-cbx ligand. This ligand has the additional possibility of C—F···H interactions, in contrast to ina. Herein, we wish to report the crystal structure of the title compound (I), which is a hydrogen bonded, three-dimensional framework.

The asymmetric unit of (I) consists of one-half of the [Zn(3-fpy4-cbx) _2_ (H_2_O) _4_] complex and a lattice water. The Zn(II) atom is located on an inversion center through which the other half of the molecular complex and another lattice water are generated from the asymmetric unit, thus completing the formula unit of (I) (Fig. 1).

The Zn(II) atom resides in a distorted ZnO_4_N_2_ octahedral environment. The equatorial positions are occupied by four O atoms from water molecules and the axial positions are occupied by N atoms from two 3-fpy4-cbx ligands. The Zn—O bond distances fall within the normal range of 2.0953 (14) - 2.1504 (13) Å (Wang *et al.*, 2006), while the Zn—N distances are also normal at 2.1356 (13) Å (Wang *et al.*, 2006). The 3-fpy4-cbx ligand is noticeably noncoplanar, with a dihedral angle of 34.2 (1)° between the mean planes of its carboxylate group and its pyridyl ring.

While the carboxylate group of 3-fpy4-cbx is not coordinated to Zn(II), it does assist in the assembly of the crystal structure by acting as hydrogen bond acceptors for both coordinated and lattice waters. The lattice waters are also involved in weak C—F···H_2_O hydrogen bonding with the 3-fpy4-cbx ligand. These interactions create a three-dimensional, hydrogen bonded network (Table 1, Fig. 2).

### Experimental

An aqueous solution of sodium 3-fluoropyridine-4-carboxylate (25 ml, 2.0 mmol) was slowly added to an aqueous solution of zinc nitrate hexahydrate (25 ml, 1.0 mmol). Colorless crystals of the title compound were obtained after slow evaporation of the resulting solution under ambient conditions.

### Refinement

All non-hydrogen atoms were refined with anisotropic displacement parameters. Hydrogen atoms bonded to carbon were placed in geometrically idealized positions and included as riding atoms: C—H = 0.93Å and with *U*_iso_(H) = 1.20-2.12 *U*_eq_(C). Oxygen-bound hydrogen atoms were located in difference Fourier maps and refined isotropically: O—H = 0.74 (2)—0.83 (3) Å and with *U*_iso_(H) = 0.94-1.69 *U*_eq_(O).

### Crystal data

**Table:**

[Zn(C_6_H_3_FNO_2_)_2_(H_2_O)_4_]·2H_2_O	*F*(000) = 464
*M**_r_* = 453.65	*D*_x_ = 1.753 Mg m^−^^3^
Monoclinic, *P*2_1_/*n*	Mo *K*α radiation, λ = 0.71073 Å
Hall symbol: -P 2yn	Cell parameters from 7697 reflections
*a* = 6.6042 (4) Å	θ = 3.0–25.0°
*b* = 19.1953 (10) Å	µ = 1.51 mm^−^^1^
*c* = 6.8697 (4) Å	*T* = 294 K
β = 99.225 (1)°	Block, colorless
*V* = 859.61 (8) Å^3^	0.28 × 0.22 × 0.16 mm
*Z* = 2	

### Data collection

**Table:**

Bruker SMART APEX CCD diffractometer	1740 independent reflections
Radiation source: fine-focus sealed tube	1605 reflections with *I* > 2σ(*I*)
graphite	*R*_int_ = 0.031
ω scans	θ_max_ = 25.0°, θ_min_ = 2.1°
Absorption correction: multi-scan (TWINABS; Bruker, 2003)	*h* = −7→7
*T*_min_ = 0.890, *T*_max_ = 1.000	*k* = 0→22
1734 measured reflections	*l* = 0→8

### Refinement

**Table:**

Refinement on *F*^2^	Primary atom site location: structure-invariant direct methods
Least-squares matrix: full	Secondary atom site location: difference Fourier map
*R*[*F*^2^ > 2σ(*F*^2^)] = 0.023	Hydrogen site location: mixed
*wR*(*F*^2^) = 0.065	H atoms treated by a mixture of independent and constrained refinement
*S* = 1.07	*w* = 1/[σ^2^(*F*_o_^2^) + (0.0427*P*)^2^ + 0.0969*P*] where *P* = (*F*_o_^2^ + 2*F*_c_^2^)/3
1740 reflections	(Δ/σ)_max_ = 0.001
149 parameters	Δρ_max_ = 0.28 e Å^−^^3^
0 restraints	Δρ_min_ = −0.19 e Å^−^^3^

### Special details

**Table:**

Geometry. All e.s.d.'s (except the e.s.d. in the dihedral angle between two l.s. planes) are estimated using the full covariance matrix. The cell e.s.d.'s are taken into account individually in the estimation of e.s.d.'s in distances, angles and torsion angles; correlations between e.s.d.'s in cell parameters are only used when they are defined by crystal symmetry. An approximate (isotropic) treatment of cell e.s.d.'s is used for estimating e.s.d.'s involving l.s. planes.
Refinement. *R*(int) value from TWINABS output.Cell_now output:Rotated from first domain by 179.9 degrees about reciprocal axis -0.159 - 0.001 1.000 and real axis -0.001 0.000 1.000Twin law to convert *hkl* from first to -1.000 0.000 - 0.317 this domain (*SHELXL* TWIN matrix): 0.001 - 1.000 0.000 - 0.002 0.000 1.000Refinement of *F*^2^ against ALL reflections. The weighted *R*-factor *wR* and goodness of fit *S* are based on *F*^2^, conventional *R*-factors *R* are based on *F*, with *F* set to zero for negative *F*^2^. The threshold expression of *F*^2^ > σ(*F*^2^) is used only for calculating *R*-factors(gt) *etc*. and is not relevant to the choice of reflections for refinement. *R*-factors based on *F*^2^ are statistically about twice as large as those based on *F*, and *R*- factors based on ALL data will be even larger.

### Fractional atomic coordinates and isotropic or equivalent isotropic displacement parameters (Å^2^)

**Table:**

	*x*	*y*	*z*	*U*_iso_*/*U*_eq_	
Zn1	0.5000	0.5000	0.5000	0.02937 (12)	
F1	0.31965 (16)	0.76809 (5)	0.48595 (19)	0.0515 (3)	
C1	0.4393 (3)	0.65492 (9)	0.4885 (2)	0.0316 (4)	
H1	0.3043	0.6403	0.4833	0.038*	
C2	0.4808 (2)	0.72491 (9)	0.4902 (2)	0.0299 (4)	
C3	0.6762 (3)	0.75042 (8)	0.4933 (2)	0.0280 (3)	
C4	0.8278 (3)	0.69967 (9)	0.4946 (3)	0.0366 (4)	
H4	0.9628	0.7131	0.4932	0.044*	
C5	0.7798 (3)	0.63022 (9)	0.4979 (3)	0.0356 (4)	
H5	0.8850	0.5978	0.5030	0.043*	
C6	0.7287 (3)	0.82734 (8)	0.5010 (3)	0.0340 (4)	
N1	0.5870 (2)	0.60704 (7)	0.4940 (2)	0.0297 (3)	
O1	0.62449 (18)	0.86516 (6)	0.5953 (2)	0.0468 (3)	
O2	0.8725 (2)	0.84601 (7)	0.4177 (2)	0.0504 (4)	
O3	0.7470 (2)	0.48608 (7)	0.7293 (2)	0.0401 (3)	
H3A	0.780 (4)	0.4489 (14)	0.789 (4)	0.068 (8)*	
H3B	0.750 (3)	0.5121 (11)	0.809 (3)	0.038 (6)*	
O4	0.6983 (2)	0.47337 (8)	0.2917 (2)	0.0413 (3)	
H4A	0.686 (4)	0.4385 (13)	0.230 (4)	0.061 (8)*	
H4B	0.708 (4)	0.5080 (12)	0.223 (4)	0.058 (8)*	
O5	1.2370 (3)	0.92014 (7)	0.5293 (2)	0.0419 (3)	
H5A	1.142 (4)	0.8983 (14)	0.505 (4)	0.068 (9)*	
H5B	1.340 (4)	0.8988 (11)	0.553 (3)	0.047 (7)*	

### Atomic displacement parameters (Å^2^)

**Table:**

	*U*^11^	*U*^22^	*U*^33^	*U*^12^	*U*^13^	*U*^23^
Zn1	0.03564 (18)	0.01479 (16)	0.03843 (19)	−0.00133 (10)	0.00817 (12)	0.00005 (10)
F1	0.0358 (6)	0.0230 (5)	0.0947 (9)	0.0041 (4)	0.0078 (6)	−0.0037 (5)
C1	0.0308 (8)	0.0223 (9)	0.0419 (10)	−0.0026 (7)	0.0064 (7)	−0.0002 (7)
C2	0.0315 (9)	0.0201 (9)	0.0383 (9)	0.0039 (6)	0.0062 (7)	−0.0004 (6)
C3	0.0341 (9)	0.0207 (8)	0.0296 (8)	−0.0022 (6)	0.0058 (7)	0.0004 (6)
C4	0.0305 (8)	0.0261 (8)	0.0546 (11)	−0.0031 (7)	0.0114 (8)	−0.0020 (8)
C5	0.0324 (9)	0.0243 (8)	0.0511 (10)	0.0016 (7)	0.0095 (8)	−0.0011 (8)
C6	0.0363 (9)	0.0222 (9)	0.0418 (10)	−0.0036 (7)	0.0011 (8)	0.0018 (7)
N1	0.0362 (8)	0.0174 (7)	0.0364 (8)	−0.0009 (6)	0.0083 (6)	0.0001 (5)
O1	0.0422 (7)	0.0245 (6)	0.0735 (9)	−0.0040 (5)	0.0089 (7)	−0.0150 (6)
O2	0.0570 (8)	0.0287 (7)	0.0701 (9)	−0.0123 (6)	0.0238 (7)	0.0029 (6)
O3	0.0514 (8)	0.0230 (7)	0.0428 (8)	0.0047 (6)	−0.0020 (6)	0.0005 (6)
O4	0.0538 (8)	0.0267 (7)	0.0481 (8)	−0.0046 (6)	0.0220 (6)	−0.0036 (7)
O5	0.0431 (8)	0.0273 (7)	0.0546 (9)	0.0025 (7)	0.0059 (7)	−0.0002 (6)

### Geometric parameters (Å, °)

**Table:**

Zn1—O3	2.0953 (14)	C4—C5	1.371 (2)
Zn1—O3^i^	2.0953 (14)	C4—H4	0.9300
Zn1—N1^i^	2.1355 (13)	C5—N1	1.345 (2)
Zn1—N1	2.1356 (13)	C5—H5	0.9300
Zn1—O4^i^	2.1504 (13)	C6—O2	1.238 (2)
Zn1—O4	2.1504 (13)	C6—O1	1.250 (2)
F1—C2	1.3457 (19)	O3—H3A	0.83 (3)
C1—N1	1.336 (2)	O3—H3B	0.74 (2)
C1—C2	1.371 (2)	O4—H4A	0.79 (3)
C1—H1	0.9300	O4—H4B	0.82 (2)
C2—C3	1.377 (2)	O5—H5A	0.75 (3)
C3—C4	1.396 (2)	O5—H5B	0.79 (2)
C3—C6	1.516 (2)		
			
O3—Zn1—O3^i^	180.0	C2—C3—C6	123.74 (15)
O3—Zn1—N1^i^	92.42 (5)	C4—C3—C6	121.34 (15)
O3^i^—Zn1—N1^i^	87.58 (5)	C5—C4—C3	120.75 (16)
O3—Zn1—N1	87.58 (5)	C5—C4—H4	119.6
O3^i^—Zn1—N1	92.42 (5)	C3—C4—H4	119.6
N1^i^—Zn1—N1	180.00 (8)	N1—C5—C4	122.80 (15)
O3—Zn1—O4^i^	90.79 (6)	N1—C5—H5	118.6
O3^i^—Zn1—O4^i^	89.21 (6)	C4—C5—H5	118.6
N1^i^—Zn1—O4^i^	91.25 (5)	O2—C6—O1	126.76 (16)
N1—Zn1—O4^i^	88.76 (5)	O2—C6—C3	117.00 (15)
O3—Zn1—O4	89.21 (6)	O1—C6—C3	116.21 (15)
O3^i^—Zn1—O4	90.79 (6)	C1—N1—C5	117.22 (15)
N1^i^—Zn1—O4	88.76 (5)	C1—N1—Zn1	117.71 (11)
N1—Zn1—O4	91.24 (5)	C5—N1—Zn1	125.06 (11)
O4^i^—Zn1—O4	180.0	Zn1—O3—H3A	126.1 (17)
N1—C1—C2	121.99 (16)	Zn1—O3—H3B	113.1 (17)
N1—C1—H1	119.0	H3A—O3—H3B	104 (2)
C2—C1—H1	119.0	Zn1—O4—H4A	122.5 (18)
F1—C2—C1	116.55 (14)	Zn1—O4—H4B	107.5 (18)
F1—C2—C3	121.15 (15)	H4A—O4—H4B	113 (3)
C1—C2—C3	122.29 (15)	H5A—O5—H5B	115 (3)
C2—C3—C4	114.90 (15)		
			
N1—C1—C2—F1	−179.40 (14)	C2—C1—N1—C5	−1.1 (2)
N1—C1—C2—C3	1.4 (3)	C2—C1—N1—Zn1	178.06 (12)
F1—C2—C3—C4	−179.08 (16)	C4—C5—N1—C1	−0.6 (3)
C1—C2—C3—C4	0.0 (2)	C4—C5—N1—Zn1	−179.72 (14)
F1—C2—C3—C6	2.6 (2)	O3—Zn1—N1—C1	−133.21 (12)
C1—C2—C3—C6	−178.23 (16)	O3^i^—Zn1—N1—C1	46.79 (12)
C2—C3—C4—C5	−1.7 (2)	O4^i^—Zn1—N1—C1	−42.37 (12)
C6—C3—C4—C5	176.61 (16)	O4—Zn1—N1—C1	137.63 (12)
C3—C4—C5—N1	2.1 (3)	O3—Zn1—N1—C5	45.92 (14)
C2—C3—C6—O2	−148.22 (17)	O3^i^—Zn1—N1—C5	−134.08 (14)
C4—C3—C6—O2	33.6 (2)	O4^i^—Zn1—N1—C5	136.77 (14)
C2—C3—C6—O1	33.4 (2)	O4—Zn1—N1—C5	−43.23 (14)
C4—C3—C6—O1	−144.78 (17)		

Symmetry codes: (i) −*x*+1, −*y*+1, −*z*+1.

### Hydrogen-bond geometry (Å, °)

**Table:**

*D*—H···*A*	*D*—H	H···*A*	*D*···*A*	*D*—H···*A*
O3—H3A···O1^ii^	0.83 (3)	1.86 (3)	2.6892 (18)	174 (2)
O3—H3B···O5^iii^	0.74 (2)	2.01 (2)	2.745 (2)	176 (2)
O4—H4A···O2^iv^	0.79 (3)	2.05 (3)	2.837 (2)	174 (2)
O4—H4B···O5^v^	0.82 (2)	1.95 (3)	2.7643 (19)	171 (3)
O5—H5A···O2	0.75 (3)	2.05 (3)	2.796 (2)	174 (3)
O5—H5B···O1^vi^	0.79 (2)	1.96 (3)	2.738 (2)	167 (2)
O5—H5B···F1^vi^	0.79 (2)	2.55 (2)	2.9929 (17)	117.2 (18)

Symmetry codes: (ii) −*x*+3/2, *y*−1/2, −*z*+3/2; (iii) *x*−1/2, −*y*+3/2, *z*+1/2; (iv) −*x*+3/2, *y*−1/2, −*z*+1/2; (v) *x*−1/2, −*y*+3/2, *z*−1/2; (vi) *x*+1, *y*, *z*.
